# *Interleukin*-17 Gene Polymorphisms Contribute to Cancer Risk

**DOI:** 10.1155/2014/128490

**Published:** 2014-07-24

**Authors:** Yu-Ming Niu, Hua Yuan, Yu Zhou

**Affiliations:** ^1^Department of Stomatology and Evidence-Based Medicine Center, Taihe Hospital, Hubei University of Medicine, 32 South Renmin Road, Shiyan 442000, China; ^2^Institute of Stomatology, Nanjing Medical University, No. 140 Hanzhong Road, Nanjing 210029, China; ^3^Department of Maxillofacial Surgery, Provincial Hospital Affiliated to Anhui Medical University, Hefei 232001, China

## Abstract

Epidemiological studies have suggested that interleukin-17 (*IL-17*) polymorphisms are associated with cancer risk. However, the results of these studies are inconsistent. Therefore, we performed a meta-analysis to obtain a precise conclusion. Odds ratios (ORs) with 95% confidence intervals (CIs) were used to assess the association of the *IL-17A* rs2275913G>A and *IL-17F* rs763780T>C polymorphisms with cancer risk. Publication bias and sensitivity analyses were performed to ensure the statistical power. Overall, 10 relevant case-control studies involving 4,516 cases and 5,645 controls were included. The pooled ORs with 95% CIs indicated that the *IL-17A* rs2275913G>A polymorphism was significantly associated with increased cancer risk (for A versus G: OR = 1.28, 95% CI: 1.16–1.41, *P* < 0.001, *I* ^2^ = 61.1%; for GA versus GG: OR = 1.12, 95% CI: 1.02–1.23, *P* = 0.015, *I* ^2^ = 27.8%; for AA versus GG: OR = 1.71, 95% CI: 1.38–2.41, *P* < 0.001, *I* ^2^ = 69.6%; for GA + AA versus GG: OR = 1.23, 95% CI: 1.13–1.34, *P* < 0.001, *I* ^2^ = 6.4%; for AA versus GG + GA: OR = 1.62, 95% CI: 1.27–2.07, *P* < 0.001, *I* ^2^ = 81.4%). Succeeding analysis of HWE and stratified analysis of gastric cancer and the Asian (and Chinese) population revealed similar results. The *IL-17F* rs763780T>C polymorphism was also significantly associated with gastric cancer development. Overall, the present meta-analysis suggests that *IL-17* polymorphisms increase the risk of developing cancer, particularly gastric cancer, in the Asian (and Chinese) population.

## 1. Introduction

Cancer is one of the most common malignancies worldwide; it is the leading cause of death in economically developed countries and the second leading cause of death in developing countries [1]. Approximately 12.7 million new cases of cancer and 7.6 million cancer-related deaths were reported in 2008 [2]. Despite the efforts exerted by many researchers to elucidate the mechanism of carcinogenesis, this process remains unclear to date. Environmental factors, diet, lifestyle, and smoking and drinking habits have been implicated in the development of cancer [3, 4]. Various epidemiological studies have revealed that inflammation-associated factors, such as interleukin- (IL-) 1, IL-6, IL-10, and tumor necrosis factor-*α*, are associated with cancer tumorigenesis [5].

Interleukin-17 (*IL-17*) is a proinflammatory cytokine that serves important functions in inflammation, autoimmune disorders, and cancer [6]. The IL-17 cytokine family consists of six members (IL-17A to IL-17F) and five receptors (IL-17RA to IL-17RD and SEF) [7, 8]. These cytokines are primarily produced from a subset of CD4+ effector cells known as Th17 cells [9, 10]. Clinical studies have shown increased IL-17 expression in malignant tumors [11–14].

Single nucleotide polymorphisms (SNPs) can alter gene functions and protein expression, which influence cell proliferation and increase cancer risk. The* IL-17A* rs2275913G>A and* IL-17F* rs763780T>C polymorphisms are the most common loci associated with* IL-17 *activity and cancer risk. In 2009, Shibata et al. [15] conducted the first study and reported a positive relationship between gastric cancer and the* IL-17A* rs2275913G>A polymorphism in a Japanese population. But no significant association was found between gastric cancer and polymorphisms of* IL-17F* rs763780 T>C. Many epidemiological studies have focused on the association of the* IL-17A* rs2275913G>A and* IL-17F* rs763780T>C polymorphisms with cancer risk. However, the results of these studies are inconsistent. Therefore, we performed a meta-analysis to clarify the possible association of the* IL-17A* rs2275913G>A and* IL-17F* rs763780T>C polymorphisms with cancer risk.

## 2. Materials and Methods 

### 2.1. Search Strategy

The PubMed, Embase, and Chinese National Knowledge Infrastructure databases were searched using the terms “cancer,” “tumor,” “interleukin-17,” “IL-17,” and “polymorphism,” (last search was updated on January 20, 2014). The “Related Articles” option was also used in each research article to find potential relevant studies on the same topic. Only studies published in English or Chinese were included. The inclusion criteria in this meta-analysis were as follows: (a) researches that focused on population, (b) studies that evaluated the association of the* IL-17A* rs2275913G>A and* IL-17F* rs763780T>C polymorphisms with cancer risk, (c) case-controls studies, and (d) studies that contain available genotype frequency to estimate the odds ratio (OR) and 95% confidence intervals (CIs). The largest or the most recent publication was selected when some data were the same or overlapped.

### 2.2. Data Extraction

Two investigators (Yu-Ming Niu and Hua Yuan) independently extracted the following information from each eligible publication: first author's name, publication year, country of origin, ethnicity of the individuals involved (categorized as Asian or Caucasian), source of controls, number of cases and controls, number of genotypes cases and controls, Hardy-Weinberg equilibrium (HWE) and minor allele frequency (MAF), and cancer category. Discrepancies were adjudicated by another author until consensus was achieved.

### 2.3. Statistical Analysis

The strength of the association of the* IL-17A* rs2275913G>A and* IL-17F* rs763780T>C polymorphisms with cancer risk was assessed by calculating crude ORs with 95% CIs. Pooled ORs were conducted for minor allele versus major allele with five models. Stratified analyses were performed by ethnicity, study design, and cancer category. Heterogeneity was calculated based on the *I* ^2^ statistic with low, moderate, and high *I* ^2^ values of 25%, 50%, and 75%, respectively [16, 17]. When *I* ^2^ ≤ 50% (which indicated a lack of heterogeneity), the OR estimation of each model was calculated by using the fixed-effects model (Mantel-Haenszel method); otherwise, the random-effects model (DerSimonian and Laird method) was used. We generated forest plots sorted by publication year. Potential publication bias was estimated using Egger's linear regression test with funnel plot [18]. Sensitivity analyses were assessed by deleting each study to reflect the influence of individual datasets on the pooled ORs [19]. Statistical analysis was performed using STATA version 11.0 (Stata Corporation, College Station, TX, USA) with two-sided *P* values. *P* < 0.05 was considered significant.

## 3. Results

### 3.1. Study Characteristics

**Figure 1:**
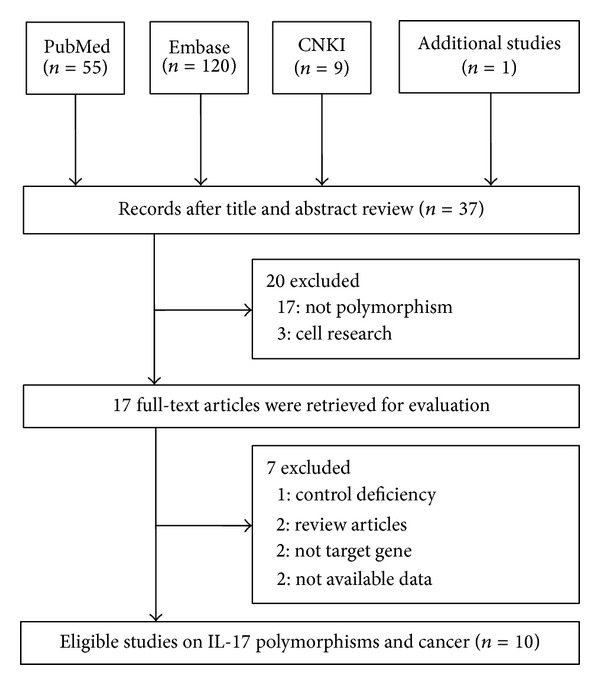
Flow chart of study selection.

A flow chart showing the study selection is presented in Figure 1. A total of 185 relevant studies were found with the research words and manual research. After careful review, 10 published case-control studies involving 4,516 cases and 5,645 controls met our inclusion criteria [15, 20–28]. We found 10 and 7 eligible studies with adequate genotype and research subjects according to* IL-17A* rs2275913G>A and* IL-17F*rs763780T>C polymorphism. All characteristics of the selected studies are summarized in Table 1. Nine studies involved Asian populations (seven involved the Chinese population), and one study involved a Caucasian population. Diverse genotyping methods were used, including polymerase chain reaction-restriction fragment length polymorphism (PCR-RFLP) [23, 27], TaqMan [21, 24, 28], polymerase chain reaction-sequence specific primers (PCR-SSCP) [15, 25], MassARRAY [20, 22], and SNaPshot SNP assay [26] methods in eligible publications. The genotypic distribution of controls in only two and three studies deviated from HWE in the* IL-17A* rs2275913G>A and* IL-17F* rs763780T>C polymorphisms, respectively.

### 3.2. Quantitative Synthesis

**Figure 2:**
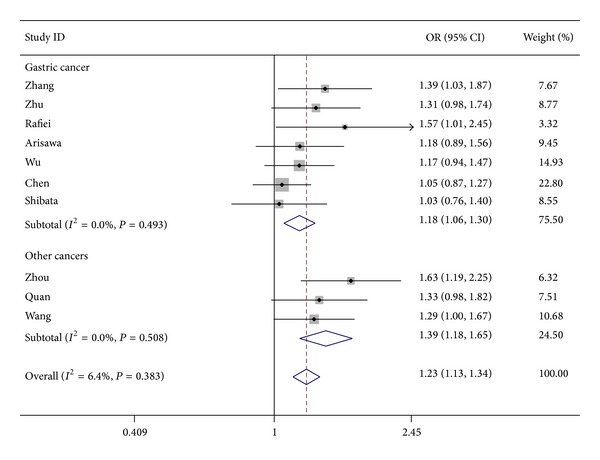
OR and 95% CIs for the association between* IL-17A* rs2275913G>A polymorphism with cancer risk for the GA + AA versus GG model.

Table 2 shows the results of this meta-analysis and the heterogeneity test. The* IL-17A* rs2275913G>A polymorphism showed significant associations with cancer risk in all populations (for A versus G: OR = 1.28, 95% CI: 1.16–1.41, *P* < 0.001, *I* ^2^ = 61.1%; for GA versus GG: OR = 1.12, 95% CI: 1.02–1.23, *P* = 0.015, *I* ^2^ = 27.8%; for AA versus GG: OR = 1.71, 95% CI: 1.38–2.41, *P* < 0.001, *I* ^2^ = 69.6%; for GA + AA versus GG: OR = 1.23, 95% CI: 1.13–1.34, *P* < 0.001, *I* ^2^ = 6.4% (Figure 2); and for AA versus GG + GA: OR = 1.62, 95% CI: 1.27–2.07, *P* < 0.001, *I* ^2^ = 81.4%). The succeeding stratified analysis according to HWE, ethnicity, and study design subgroup also presented that the* IL-17A* rs2275913G>A polymorphism may be a strong risk factor in the development of cancer, especially gastric cancer, in the Chinese population.

**Figure 3:**
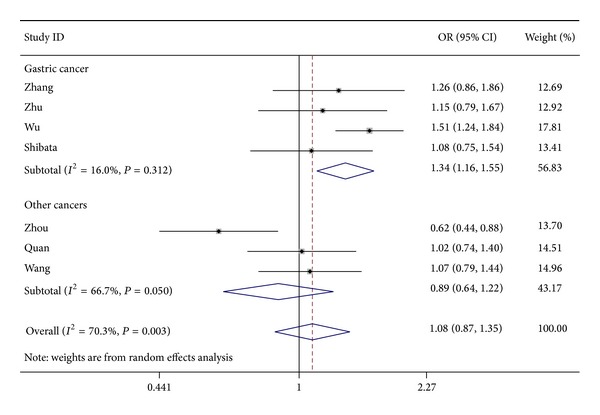
OR and 95% CIs for the association between* IL-17F* rs763780T>C polymorphisms and cancer risk in TC + CC versus TT model.

Statistical analysis also indicated that the* IL-17F* rs763780T>C polymorphisms were significantly associated with cancer risk, particularly gastric cancer (for C versus T: OR = 1.29, 95% CI: 1.14–1.46, *P* < 0.001, *I* ^2^ = 0%; for TC versus TT: OR = 1.33, 95% CI: 1.13–1.55, *P* < 0.001, *I* ^2^ = 21.6%; for CC versus TT: OR = 1.40, 95% CI: 1.04–1.88, *P* = 0.026, *I* ^2^ = 0%; for TC + CC versus TT: OR = 1.34, 95% CI: 1.16–1.55, *P* < 0.001, *I* ^2^ = 16% (Figure 3)).

### 3.3. Sensitivity Analysis

**Figure 4:**
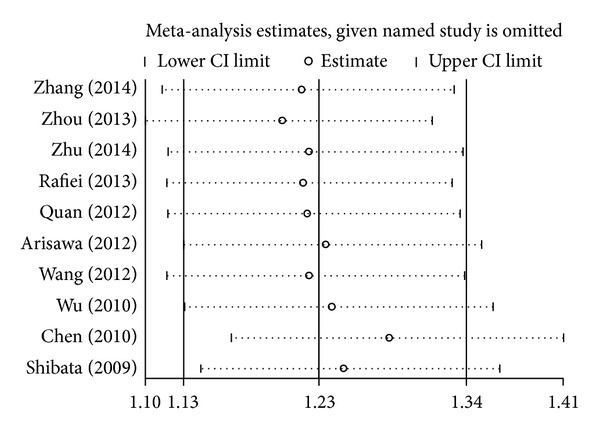
Sensitivity analysis through deleting each study to reflect the influence of the individual dataset on the pooled ORs in GA + AA versus GG model of* IL-17A* rs2275913G>A polymorphism.

**Figure 5:**
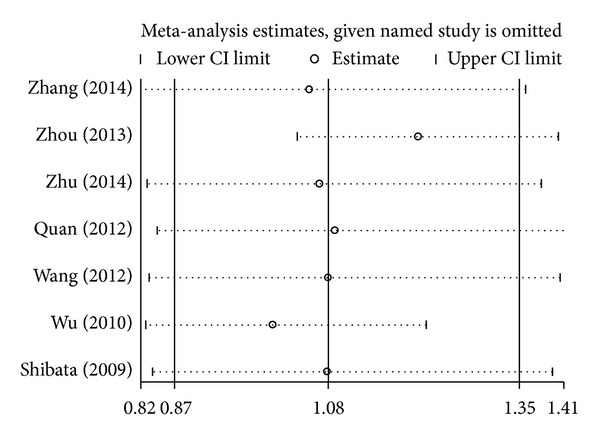
Sensitivity analysis through deleting each study to reflect the influence of the individual dataset on the pooled ORs in TC + CC versus TT model of* IL-17F* rs763780T>C polymorphism.

A single study involved in the meta-analysis was deleted each time to reflect the influence of the individual data set on the pooled ORs, and the corresponding pooled ORs were not qualitatively altered. This indicated that the results about the association between* IL-17* gene polymorphisms and cancer risk were statistically robust (Figures 4 and 5).

### 3.4. Publication Bias

**Figure 6:**
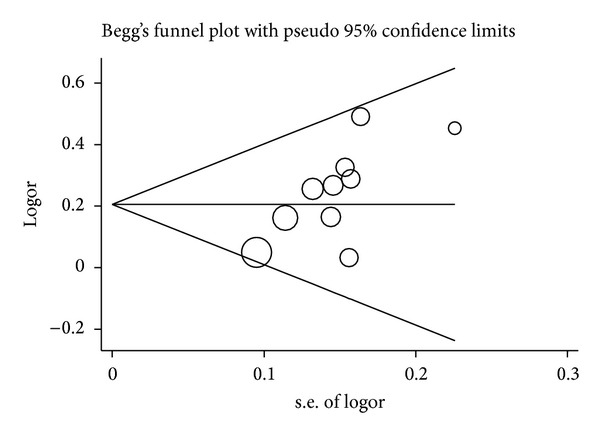
Funnel plot analysis to detect publication bias for GA + AA versus GG model of* IL-17A* rs2275913G>A polymorphism. Each point represents a separate study.

**Figure 7:**
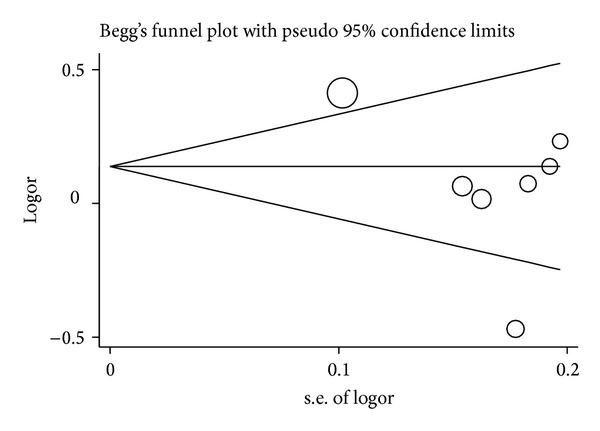
Funnel plot analysis to detect publication bias for TC + CC versus TT model of* IL-17F* rs763780T>C polymorphism. Each point represents a separate study.

Funnel plot and Egger's test were performed to estimate the publication bias of literature. Publication bias was detected in the meta-analyses on the allele contrast, homozygote (AA versus GG), dominant, and recessive models of the* IL-17A* rs2275913G>A polymorphism (Figure 6 for GA + AA versus GG model), except for the GA versus GG model (*P* = 0.652). Stratified analyses were conducted only with the HWE and Chinese population, but the results were not substantially different. For the* IL-17F* rs763780T>C polymorphism, the funnel plots did not show any asymmetrical evidence in all genetic models (Figure 7). The result was further supported by analysis using Egger's tests (*P* = 0.102 for C versus T; *P* = 0.185 for TC versus TT; *P* = 0.382 for CC versus TT; *P* = 0.114 for TC + CC versus TT (Figure 7); *P* = 0.792 for CC versus TT + TC).

## 4. Discussion

Carcinogenesis is a multistep process that involves numerous factors, such as smoking, drinking, xenobiotics infections, nutrition deficiency, and host genetic factor. Cancer-related inflammation factors have been recently confirmed to increase the risk of developing malignant tumors. IL-17 is a relatively novel cytokine family that is connected with adaptive and innate immune systems. IL-17A and IL-17F are members of the IL-17 cytokine family that are responsible for the pathogenic activity of IL-17 cells, the lineage of CD4^+^ effector cells, and multiple proinflammatory mediators [29].

Genetic polymorphisms of the IL-17A and IL-17F cytokines could change the function and expression of cytokines, which influence the activity of ILs [12, 30, 31]. Several studies have revealed that* IL-17A* and* IL-17F* polymorphisms are associated with gastric cancer, breast cancer, and so on. However, the results of these studies are inconsistent.

In 2009, Shibata et al. [15] were the first to report that the AA homozygote was significantly correlated with the development of gastric cancer compared with the common homozygous genotype (GG) in a Japanese population (OR = 3.02, 95% CI: 1.86–4.91). One year later, Chen et al. [28] also found a positive relationship between gastric cancer and the* IL-17A* rs2275913G>A polymorphism in a Chinese Han population with a drinking habit (for A versus G: OR = 1.37, 95% CI: 1.07–1.76). Similar results were reported by Arisawa et al. [25], Rafiei et al. [23], Zhang et al. [20], and Zhuet al. [22] in gastric cancer. Furthermore, the mutation of* IL-17A* rs2275913G>A locus was also demonstrated as tumorigenic for bladder cancer [21], breast cancer [26], and cervical cancer [24]. However, another article detected no significant association between the* IL-17A *rs2275913G>A polymorphism and gastric cancer risk [27].

Regarding the* IL-17F* rs763780T>C polymorphism, Wu et al. [27] found that the CT and CC genotypes are associated with an increased risk of gastric cancer compared with the TT genotype (OR = 1.51, 95% CI: 1.22–1.87 for CT; OR = 1.61, 95% CI: 1.03–2.51 for CC). By contrast, Zhou et al. [21] showed that bladder cancer patients have significantly higher frequencies of T allele than controls. This result indicates that this T allele is significantly associated with bladder cancer (OR = 1.46, 95% CI: 1.07–2.00). Furthermore, other researches did not find any significant association between the* IL-17F* rs763780T>C polymorphism and cancer risk [15, 20, 22, 24, 26].

To the best of our knowledge, this meta-analysis is the first to determine the association of* IL-17* polymorphisms with cancer risk. This study focused on two common* IL-17* polymorphisms, namely,* IL-17A* rs2275913G>A (10 studies with 4,516 cases and 5,645 controls) and* IL-17F* rs763780T>C (7 studies with 2,863 cases and 3,773 controls). Significant associations were found between the* IL-17A* rs2275913G>A polymorphism and cancer risk in all five genotype models of total populations. Besides, we also detected some association between the* IL-17F* rs763780T>C polymorphism and the risk of Asians (Chinese), population-based and hospital-based controls, and gastric cancer or other cancers in the subgroup analysis by HWE publications, ethnicity, control design, and cancer category. Interestingly, nine researches focused on Asian population; the results of our meta-analysis demonstrated that the* IL-17A* rs2275913G>A polymorphism may be a stranger canner risk for Asian ethnicity (including Chinese). Moreover, the category of control design did not influence the results; not only the population-based but also the hospital-based controls all showed that significant association existed between* IL-17A* rs2275913G>A polymorphism and cancer risk. In all selected publications, seven studies focused on gastric cancer and the results also indicated that* IL-17A* rs2275913G>A polymorphism plays an important role during the development of gastric cancer. For* IL-17F* rs763780T>C polymorphism, all selected studies were conducted in Asian population and significant association only was found in codominant model (CC versus TT) in total population. Furthermore, subgroup analyses revealed a significantly increased risk of gastric cancer with* IL-17F* rs763780T>C polymorphism in four models. Another stratified analysis of population-based control also drew a consistent conclusion.

This meta-analysis has several limitations in result interpretation. First, each gene only has a moderate effect on cancer development. A combination of relative genotypes may be a higher risk factor than a single locus genotype. Linkage disequilibrium and haplotype analyses of the two polymorphisms were not conducted because of the lack of original data on the individual genotypes from the included studies. Second, certain publication biases existed until subgroup analyses were conducted. These deviations would influence the correctness and reliability of the results. Third, these results were based on unadjusted estimates, and the evaluations were limited without the effects of gene-gene and gene-environment interactions. Finally, most of the included studies had been conducted on Asians but not on Caucasians and Africans, and the association between ethnicity variation and cancer risk could not be explored deeply.

In conclusion, despite these limitations, the present meta-analysis demonstrates that the* IL-17A* rs2275913G>A polymorphism is associated with cancer development. Furthermore, the* IL-17F* rs763780T>C polymorphism may be a potential risk factor in the development of gastric cancer. In the future, large-scale, case-control, and well-designed studies must be conducted to validate the findings of our meta-analysis and to comprehensively understand the potential gene-gene and gene-environment interactions between* IL-17* polymorphisms and cancer risk.

## Figures and Tables

**Table 1:** Characteristics of case-control studies on IL-17A rs2275913G>A and IL-17F rs763780 T>C polymorphisms and cancer risk included in the meta-analysis.

First author	Year	Country	Ethnicity	Source of controls	Case	Control	Genotype distribution						*P *for HWE^a^	MAF	Genotyping method	Cancer category
							Case			Control		
							G/G	G/A	A/A	G/G	G/A	A/A
Zhang [20]	2014	China	Asian	Population controls	260	512	110	102	48	258	187	67	<0.01	0.31	MassARRAY	Gastric cancer
Zhou [21]	2013	China	Asian	Hospital controls	301	446	79	154	68	164	204	78	0.29	0.40	TaqMan	Bladder cancer
Zhu [22]	2014	China	Asian	Hospital controls	293	550	126	122	45	273	216	61	0.07	0.31	MassARRAY	Gastric cancer
Rafiei [23]	2013	Iran	Caucasian	Healthy controls	161	171	56	61	44	78	72	21	0.49	0.33	PCR-RFLP	Gastric cancer
Quan [24]	2012	China	Asian	Hospital controls	311	463	93	142	76	168	215	80	0.43	0.40	TaqMan	Cervical cancer
Arisawa [25]	2012	Japan	Asian	Hospital controls	333	583	112	137	84	218	293	72	0.08	0.37	PCR-SSCP	Gastric cancer
Wang [26]	2012	China	Asian	Population controls	491	501	165	234	92	198	245	58	0.17	0.36	SNaPshot SNP assay	Breast cancer
Wu [27]	2010	China	Asian	Population controls	945	768	210	485	250	193	371	204	0.35	0.51	PCR-RFLP	Gastric cancer
Chen [28]	2010	China	Asian	Population controls	1042	1090	300	522	220	325	541	224	0.97	0.45	TaqMan	Gastric cancer
Shibata [15]	2009	Japan	Asian	Hospital controls	287	523	94	124	69	175	299	49	<0.01	0.38	PCR-SSCP	Gastric cancer

							T/T	T/C	C/C	T/T	T/C	C/C				

Zhang [20]	2014	China	Asian	Population controls	260	512	209	30	21	429	53	30	<0.01	0.11	MassARRAY	Gastric cancer
Zhou [21]	2013	China	Asian	Hospital controls	301	446	240	57	4	317	124	5	0.06	0.15	TaqMan	Bladder cancer
Zhu [22]	2014	China	Asian	Hospital controls	293	550	241	35	17	463	58	29	<0.01	0.11	MassARRAY	Gastric cancer
Quan [24]	2012	China	Asian	Hospital controls	311	462	222	85	4	332	126	5	0.06	0.15	TaqMan	Cervical cancer
Wang [26]	2012	China	Asian	Population controls	491	502	382	103	6	396	99	7	0.77	0.11	SNaPshot SNP assay	Breast cancer
Wu [27]	2010	China	Asian	Population controls	927	777	540	332	55	527	214	36	0.02	0.18	PCR-RFLP	Gastric cancer
Shibata [15]	2009	Japan	Asian	Hospital controls	280	523	221	55	4	419	100	4	0.46	0.10	PCR-SSCP	Gastric cancer

^a^HWE in control.

MAF: minor allele frequency in control group.

**Table 2:** Summary of ORs and 95% CI of IL-17A rs2275913G>A and IL-17F rs763780T>C polymorphisms and cancer risk.

	*N* ∗	A versus G				GA versus GG				AA versus GG				GA + AA versus GG				AA versus GG + GA			
		OR	95% CI	*P *	*I* ^2^ (%)^a^	OR	95% CI	*P *	*I* ^2^ (%)^a^	OR	95% CI	*P *	*I* ^2^ (%)^a^	OR	95% CI	*P *	*I* ^2^ (%)^a^	OR	95% CI	*P *	*I* ^2^ (%)^a^
Total	10	**1.28**	**1.16–1.41**	**<0.001**	**61.1**	**1.12**	**1.02–1.23**	**0.015**	**27.8**	**1.71**	**1.38–2.14**	**<0.001**	**69.6**	**1.23**	**1.13–1.34**	**<0.001**	**6.4**	**1.62**	**1.27–2.07**	**<0.001**	**81.4**
HWE	8	**1.26**	**1.13–1.41**	**<0.001**	**66.4**	**1.15**	**1.04–1.27**	**0.008**	**0**	**1.63**	**1.29–2.08**	**<0.001**	**70**	**1.23**	**1.12–1.36**	**<0.001**	**9.7**	**1.51**	**1.18–1.93**	**0.001**	**78.4**
Ethnicity																					
Asian	9	**1.25**	**1.14–1.37**	**<0.001**	**56.5**	**1.12**	**1.02–1.23**	**0.019**	**35.6**	**1.65**	**1.32–2.05**	**<0.001**	**68.6**	**1.22**	**1.12–1.33**	**<0.001**	**4.4**	**1.55**	**1.21–1.99**	**0.001**	**81.6**
China	7	**1.21**	**1.10–1.34**	**<0.001**	**56.2**	**1.19**	**1.07–1.32**	**0.001**	**0**	**1.46**	**1.20–1.79**	**<0.001**	**54.3**	**1.24**	**1.13–1.37**	**<0.001**	**14.9**	**1.30**	**1.09–1.56**	**0.004**	**56.6**
Design																					
PB	4	**1.16**	**1.01–1.32**	**0.031**	**63.3**	**1.14**	**1.01–1.29**	**0.041**	**0**	**1.34**	**1.02–1.76**	**0.033**	**65**	**1.18**	**1.05–1.32**	**0.006**	**1.1**	1.23	0.96–1.59	0.108	69.7
HB	5	**1.35**	**1.23–1.48**	**<0.001**	**0**	1.10	0.87–1.39	0.447	63.1	**1.97**	**1.64–2.37**	**<0.001**	**0**	**1.28**	**1.12–1.46**	**<0.001**	**11.8**	**1.87**	**1.38–2.52**	**<0.001**	**69.2**
Location																					
Gastric	7	**1.26**	**1.11–1.44**	**<0.001**	**70**	1.07	0.96–1.19	0.202	24.2	**1.70**	**1.26–2.30**	**0.001**	**77.8**	**1.18**	**1.06–1.30**	**0.001**	**0**	**1.67**	**1.18–2.36**	**0.004**	**87**
Others	3	**1.33**	**1.19–1.49**	**<0.001**	**0**	**1.27**	**1.05–1.53**	**0.013**	**6.6**	**1.81**	**1.43–2.29**	**<0.001**	**0**	**1.39**	**1.18–1.65**	**<0.001**	**0**	**1.56**	**1.27–1.91**	**<0.001**	**0**

		C versus T				TC versus TT				CC versus TT				TC + CC versus TT				CC versus TT + TC			

Total	7	1.09	0.91–1.30	0.347	64.6	1.06	0.84–1.34	0.629	70	**1.33**	**1.02–1.75**	**0.038**	**0**	1.08	0.87–1.35	0.486	70.3	1.26	0.96–1.65	0.098	0
HWE	4	0.95	0.81–1.10	0.516	46.4	0.92	0.71–1.19	0.650	57.2	1.15	0.61–2.17	0.660	0	0.93	0.72–1.19	0.557	55.5	1.18	0.62–2.21	0.616	0
Ethnicity																					
China	6	1.08	0.88–1.33	0.439	70.4	1.06	0.81–1.39	0.676	74.7	**1.32**	**1.00–1.74**	**0.053**	**0**	1.08	0.84–1.39	0.555	75.1	1.24	0.94–1.63	0.130	0
Design																					
PB	3	**1.29**	**1.13–1.41**	**<0.001**	**32.4**	**1.34**	**1.14–1.57**	**<0.001**	**43.4**	**1.40**	**1.01–1.96**	**0.045**	**0**	**1.34**	**1.05–1.57**	**<0.001**	**45.8**	1.29	0.92–1.79	0.136	0
HB	4	0.97	0.77–1.21	0.550	52.2	0.92	0.69–1.22	0.554	57.3	1.20	0.74–1.94	0.466	0	0.84	0.72–1.23	0.643	58.1	1.20	0.74–1.94	0.456	0
Location																					
Gastric	4	**1.29**	**1.14–1.46**	**<0.001**	**0**	**1.33**	**1.13–1.55**	**<0.001**	**21.6**	**1.40**	**1.04–1.88**	**0.026**	**0**	**1.34**	**1.16–1.55**	**<0.001**	**16**	1.30	0.97–1.74	0.082	0
Others	3	0.91	0.70–1.18	0.472	57.4	0.88	0.62–1.24	0.460	69.2	1.02	0.50–2.08	0.962	0	0.89	0.64–1.22	0.461	66.7	1.04	0.51–2.13	0.905	0

*Numbers of comparisons.

^a^Test for heterogeneity.
